# Mongooses (*Urva auropunctata*) as reservoir hosts of *Leptospira* species in the United States Virgin Islands, 2019–2020

**DOI:** 10.1371/journal.pntd.0009859

**Published:** 2021-11-15

**Authors:** Hannah M. Cranford, A. Springer Browne, Karen LeCount, Tammy Anderson, Camila Hamond, Linda Schlater, Tod Stuber, Valicia J. Burke-France, Marissa Taylor, Cosme J. Harrison, Katia Y. Matias, Alexandra Medley, John Rossow, Nicholas Wiese, Leanne Jankelunas, Leah de Wilde, Michelle Mehalick, Gerard L. Blanchard, Keith R. Garcia, Alan S. McKinley, Claudia D. Lombard, Nicole F. Angeli, David Horner, Thomas Kelley, David J. Worthington, Jennifer Valiulis, Bethany Bradford, Are Berentsen, Johanna S. Salzer, Renee Galloway, Ilana J. Schafer, Kristine Bisgard, Joseph Roth, Brett R. Ellis, Esther M. Ellis, Jarlath E. Nally

**Affiliations:** 1 Virgin Islands Department of Health, Epidemiology Division, Christiansted, Virgin Islands, United States of America; 2 Epidemic Intelligence Service, Division of Scientific Education and Professional Development, Centers for Disease Control and Prevention, Atlanta, Georgia, United States of America; 3 Leptospira Working Group, National Centers for Animal Health, United States Department of Agriculture, Ames, Iowa, United States of America; 4 National Veterinary Services Laboratories, Animal and Plant Health Inspection Service, United States Department of Agriculture, Ames, Iowa, United States of America; 5 Virgin Islands Department of Health, Public Health Laboratory, Christiansted, Virgin Islands, United States of America; 6 Laboratory Leadership Service, Centers for Disease Control and Prevention, Atlanta, Georgia, United States of America; 7 Epidemiology Elective Program, Centers for Disease Control and Prevention, Atlanta, Georgia, United States of America; 8 St. Croix Animal Welfare Center, Christiansted, Virgin Islands, United States of America; 9 Animal and Plant Health Inspection Service Wildlife Services, United States Department of Agriculture, Charlotte Amalie, Virgin Islands, United States of America; 10 United States Fish and Wildlife Service, Christiansted, Virgin Islands, United States of America; 11 United States Virgin Islands Department of Planning and Natural Resources, Christiansted, Virgin Islands, United States of America; 12 National Park Service, Cruz Bay, Virgin Islands, United States of America; 13 St. Croix Environmental Association, Christiansted, Virgin Islands, United States of America; 14 United States Virgin Islands Department of Agriculture, Christiansted, Virgin Islands, United States of America; 15 Animal and Plant Health Inspection Service Wildlife Services, National Wildlife Research Center, United States Department of Agriculture, Ames, Iowa, United States of America; 16 Bacterial Special Pathogens Branch, Division of High-Consequence Pathogens and Pathology, Centers for Disease Control and Prevention, Atlanta, Georgia, United States of America; 17 Center for Surveillance, Epidemiology, and Laboratory Services, Centers for Disease Control and Prevention, Atlanta, Georgia, United States of America; 18 Agricultural Research Service, Infectious Bacterial Diseases Research Unit, United States Department of Agriculture, Ames, Iowa, United States of America; Faculty of Medicine and Health Sciences, Universiti Putra Malaysia, MALAYSIA

## Abstract

During 2019–2020, the Virgin Islands Department of Health investigated potential animal reservoirs of *Leptospira* spp., the bacteria that cause leptospirosis. In this cross-sectional study, we investigated *Leptospira* spp. exposure and carriage in the small Indian mongoose (*Urva auropunctata*, syn: *Herpestes auropunctatus*), an invasive animal species. This study was conducted across the three main islands of the U.S. Virgin Islands (USVI), which are St. Croix, St. Thomas, and St. John. We used the microscopic agglutination test (MAT), fluorescent antibody test (FAT), real-time polymerase chain reaction (*lipl32* rt-PCR), and bacterial culture to evaluate serum and kidney specimens and compared the sensitivity, specificity, positive predictive value, and negative predictive value of these laboratory methods. Mongooses (n = 274) were live-trapped at 31 field sites in ten regions across USVI and humanely euthanized for *Leptospira* spp. testing. Bacterial isolates were sequenced and evaluated for species and phylogenetic analysis using the *ppk* gene. Anti-*Leptospira* spp. antibodies were detected in 34% (87/256) of mongooses. Reactions were observed with the following serogroups: Sejroe, Icterohaemorrhagiae, Pyrogenes, Mini, Cynopteri, Australis, Hebdomadis, Autumnalis, Mankarso, Pomona, and Ballum. Of the kidney specimens examined, 5.8% (16/270) were FAT-positive, 10% (27/274) were culture-positive, and 12.4% (34/274) were positive by rt-PCR. Of the *Leptospira* spp. isolated from mongooses, 25 were *L*. *borgpetersenii*, one was *L*. *interrogans*, and one was *L*. *kirschneri*. Positive predictive values of FAT and rt-PCR testing for predicting successful isolation of *Leptospira* by culture were 88% and 65%, respectively. The isolation and identification of *Leptospira* spp. in mongooses highlights the potential role of mongooses as a wildlife reservoir of leptospirosis; mongooses could be a source of *Leptospira* spp. infections for other wildlife, domestic animals, and humans.

## Author summary

Leptospirosis is a zoonotic disease caused by bacteria of the genus *Leptospira*. To better understand local reservoirs and risk factors to humans and animals, during 2019–2020, the Virgin Islands Department of Health (VIDOH) investigated *Leptospira* spp. in association with the small Indian mongoose (*Urva auropunctata*, syn: *Herpestes auropunctatus*) across the three main islands of the United States Virgin Islands (USVI) (St. Croix, St. Thomas, and St. John). Mongoose blood and kidney samples were evaluated by laboratory testing: microscopic agglutination test, fluorescent antibody test (FAT), real-time polymerase chain reaction (*lipl32* rt-PCR), and culture. Of 256 mongooses, 34% were exposed to *Leptospira* spp. To assess active infection in mongooses, kidney samples were examined resulting 5.8% (16/270) FAT-positive, 10% (27/274) culture-positive, and 12.4% (34/274) rt-PCR-positive. Validity was compared between test types showing higher positive predictive value for FAT and rt-PCR testing for predicting successful culture, the reference standard for diagnosis. Through the detection and isolation of *Leptospira* spp. in mongooses across the USVI, we identified mongooses as potential disease vectors to the local population and added to the limited data for the Caribbean region. Furthermore, a validity assessment of reference testing provides data to potentially inform future leptospirosis diagnostics.

## Introduction

Leptospirosis is an infectious disease caused by diderm bacteria of the genus *Leptospira* with an estimated 1.03 million cases and 58,900 deaths annually worldwide [1]. *Leptospira* spp. are maintained in animal hosts and transmitted to humans through direct animal contact and environmental exposure to water and soil contaminated by the urine of infected animals. Most cases in the United States have occurred in tropical and subtropical areas [2].

The United States Virgin Islands (USVI) is a territory of the United States of America, located in the Caribbean region, approximately 40 miles east of Puerto Rico. The main islands of St. Croix, St. John, and St. Thomas, along with surrounding minor islands, form a total land area of 133 square miles (344 square kilometers), with an estimated human population in 2010 of 106,405 people [3]. After the 2017 Hurricanes Irma and Maria, the Virgin Islands Department of Health (VIDOH) identified the first three cases of human leptospirosis documented in the USVI [4]; hurricane events can lead to increased transmission of leptospirosis due to human exposure to floodwaters [5]. A 2019 cross-sectional serosurvey in USVI detected exposure to *Leptospira* spp. in an unweighted 3.9% of people sampled (n = 1206) [6]. Animals have previously been shown to be serologically positive to *Leptospira* spp. in the USVI, including goats on St. Croix (26%, 28/108) for seven *Leptospira* serogroups (Autumnalis, Ballum, Bataviae, Australis, Canicola, Icterohaemorrhagiae and Pyrogenes), and sheep (32%, 17/53) for the same seven serogroups plus serogroup Sejroe (sv Hardjo) [7]. During 2011–2017, two of five serum specimens from white-tailed deer on St. John had detectable antibody titers to *Leptospira* spp. [8]. After VIDOH’s identification of leptospirosis in USVI following Hurricanes Irma and Maria, and given limited data on animal reservoirs in USVI, animal leptospirosis surveillance projects that included rodents, livestock, mongooses, dogs, and bats were led by VIDOH with the help of local and federal collaborators. One such project, implemented during 2019–2020, identified 37.6% (47/125) of livestock sampled in St. Croix, USVI, as seropositive for *Leptospria* spp., with urine from 4 animals (4/101) positive by real-time polymerase chain reaction (*lipl32* rt-PCR). [9]. These surveillance projects initiated a dramatic increase in laboratory and logistical testing capacity for USVI and will inform the public health response to human leptospirosis infections in the USVI community through targeted messaging, outreach, and potential public health interventions.

The small Indian mongoose (*Urva auropunctata*) is an invasive species in USVI that was introduced in the late 19^th^ century throughout the Caribbean region to control rodent populations in sugarcane fields [10]. Mongooses have been identified as potential reservoirs of *Leptospira* spp. in other tropical regions: 13% (12/89) of mongooses had *Leptospira* spp. detected by *lipl32* rt-PCR in Puerto Rico [11]; 6.7% (9/146) tested positive by *lipl32* rt-PCR and two culture positives were identified as *L*. *interrogans* serogroup Icterohaemorrhagiae in St. Kitts [12]; and four positive *Leptospira* spp. cultures *(2.9%, 95% CI 0.8, 7.4)* were obtained in Barbados *[13]*. Following serological testing in St. Kitts, leptospirosis seroprevalence was 8.1% (12/148), with primary serum reactivity to serogroup Mankarso [12]. However in Barbados, seroprevalence was 40.7% (48/118) with primary serum reactivity to serogroup Autumnalis [13]. Mongooses have not been investigated as a potential reservoir for leptospirosis in USVI.

Through this project, the VIDOH maintained two objectives: (a) investigate mongooses as potential leptospirosis reservoirs using seroprevalence (exposure), kidney presence of bacteria (fluorescent antibody test [FAT] and *lipl32* rt-PCR testing), and bacterial culture of *Leptospira* spp. and (b) use such extensive testing methodology to compare common screening methods against the gold standard of bacterial culture, by evaluating the sensitivity, specificity, positive predictive value, and negative predictive value.

## Methods

### Ethics and permits

Sampling procedures for mongooses were approved by the Centers for Disease Control and Prevention (CDC) Institutional Animal Care and Use Committee (IACUC), under protocol number 2929DOTMULX-A5. This protocol details required field practices, including requirements for anesthesia, and is in line with American Veterinary Medical Association (AVMA) guidelines for humane euthanasia [14]. All personnel handling mongooses were vaccinated for rabies virus within the previous year; titer checks were performed on all local collaborators who had been previously vaccinated >1 year before field work commenced. Sampling permits were obtained from the National Park Service, U.S. Fish & Wildlife Service, and USVI Department of Planning and Natural Resources.

### Sampling methods

We selected mongoose sampling regions based on contiguous ecological areas, which differed by island but covered the entire area of each island; four sampling regions were identified on St. Croix, three on St. Thomas, and three on St. John. Target population in each sampling unit was the immediate population of mongooses within each of the 10 regions. Based on laboratory and field resources, a minimum of 24 mongooses were targeted to be captured in each of the 10 regions. We trapped live mongooses in 6 × 6 × 18-inch (15 × 15 × 46 cm) Tomahawk traps baited with Vienna sausage. Island, habitat, and GPS coordinates for each trap were recorded, in addition to age and sex for each mongoose trapped. Mongooses were put under a deep plane of anesthesia and humanely euthanized using recommended protocols and AVMA guidelines for humane euthanasia [14].

Blood was collected from the heart and placed in serum separator tubes (7.5 mL volume). We then immediately conducted a field necropsy and collected one kidney using sterile technique, placing it in liquid Hornsby-Alt-Nally (HAN) media containing 5-Fluoruracil (5-FU, 100ug/ml) for *Leptospira* culture [15]. The second kidney was placed in a sterile bag for rt-PCR testing. All samples were kept on ice in the field. At the VIDOH Laboratory, serum was separated within 12 hours of collection and then samples were stored in a -80°C freezer until shipping to either CDC’s Bacterial Special Pathogens Branch laboratory (CDC-BSPB) (Atlanta, Georgia, USA) or USDA’s National Veterinary Service Laboratories (NVSL) (Ames, Iowa, USA) for rt-PCR and microscopic agglutination testing (MAT). All kidneys in HAN media were shipped overnight to USDA’s Agricultural Research Service-National Animal Disease Center (ARS-NADC) for FAT and culture.

## Laboratory methods

### Microscopic agglutination testing (MAT)

For the detection of anti-*Leptospira* antibodies in mongoose sera, we performed MAT as previously described [16] and according to World Organisation for Animal Health guidelines [17], using a panel of either 18 antigens, representing 15 serogroups (NVSL) or 20 antigens, representing 17 serogroups (CDC-BSPB) (S1 Table). MAT positivity was defined as any titer ≥1:100.

### *Leptospira* spp. detection

We used FAT, rt-PCR, and culture to detect *Leptospira* spp. in mongoose kidney. FAT was performed as described [18]. DNA was extracted from manually macerated kidney homogenate using the Maxwell RSC Purefood Purification Pathogen kit (NVSL) or whole blood DNA kit (CDC) (Promega Corporation, Madison, Wisconsin), and rt-PCR was performed to detect the *lipl32* gene (found only in pathogenic *Leptospira* spp.) as described [19,20].

For *Leptospira* culture, we shipped whole mongoose kidneys in 50ml conical tubes with 10ml liquid HAN medium. Kidneys were transferred to a 150mm petri dish. The capsule was removed, and the kidney transferred to a 710ml Whirl-Pak (World Bioproducts, LLC, Madison, Wisconsin) bag. Ten ml of liquid HAN was added, and tissue was macerated manually. Two 10-fold serial dilutions were made from the macerate in liquid HAN and 200μl of the 10^2^ dilution was used to inoculate one tube each: modified T80/40/LH semisolid medium [21], BA-P-80 semisolid medium (NVSL, Ames, IA), HAN semisolid and HAN liquid media [15]. One tube of modified T80/40/LH semisolid medium was inoculated with 200μl of the 10^3^ dilution. T80/40/LH and BA-P-80 tubes were incubated at 29°C, HAN tubes were incubated at 37°C in 3% CO_2_. Per USDA-NVSL protocol, tubes were observed for growth for six months before being considered negative.

### Serotyping and molecular characterization of *Leptospira* spp.

Isolates of *Leptospira* spp. cultured from mongooses were serotyped by MAT, with a panel of polyclonal rabbit reference antisera representing the same 15 serogroups used in the NVSL MAT panel (S1 Table). DNA from each isolate was isolated using the Maxwell RSC Purefood Purification Pathogen kit (Promega Corporation, Madison, Wisconsin) according to manufacturer’s guidelines. Concentration of reconstituted genomic DNA was determined by Qubit (Qubit dsDNA BR assay, Qubit 3.0 fluorometer, Invitrogen), and gDNA libraries were prepared using the Nextera XT DNA Library Preparation Kit (Ilumina, Inc., San Diego, California) per manufacturer’s instructions. Sequencing was performed using 2×250 Cycle v2 chemistry on a MiSeq Desktop Sequencer per manufacturer’s instructions. Draft assemblies of each genome were mined to retrieve the full length *ppk* coding regions. The *ppk* coding regions were used to determine *Leptospira* species using the Institut Pasteur *Leptospira* Sequence Database [22]. A RaxML phylogenetic tree was constructed using an alignment of the *ppk* gene; Vincent et al. determined the *ppk* gene to be highly effective at genomic comparison for *Leptospira*, similar to softcore gene methods [23,24].

### Statistical analysis

Mapping was performed using R (package version 4.0.2) [25]. To assess the effectiveness of common laboratory screening methods for *Leptospira*, sensitivity, specificity, positive predictive value, and negative predictive value calculations were performed on rt-PCR, FAT, and MAT testing results, using bacterial culture as a gold standard. Summary statistics were performed for prevalence calculations, and the R 4.0.2 package “iccbin” was used to determine intraclass correlation coefficients (*ρ*) using a Monte Carlo 1-way generalized linear mixed model for each laboratory method, by mongoose location and island [25,26]. A description of the strength of correlation is as follows: 0.00–0.19: “very weak”; 0.20–0.39: “weak”; 0.40–0.59: “moderate”; 0.60–0.79: “strong”; and 0.80–1.0: “very strong”. A *ppk* gene phylogenetic tree was illustrated and annotated using Interactive Tree of Life version 5.7 (itol.dmbl.de).

## Results

**Fig 1:**
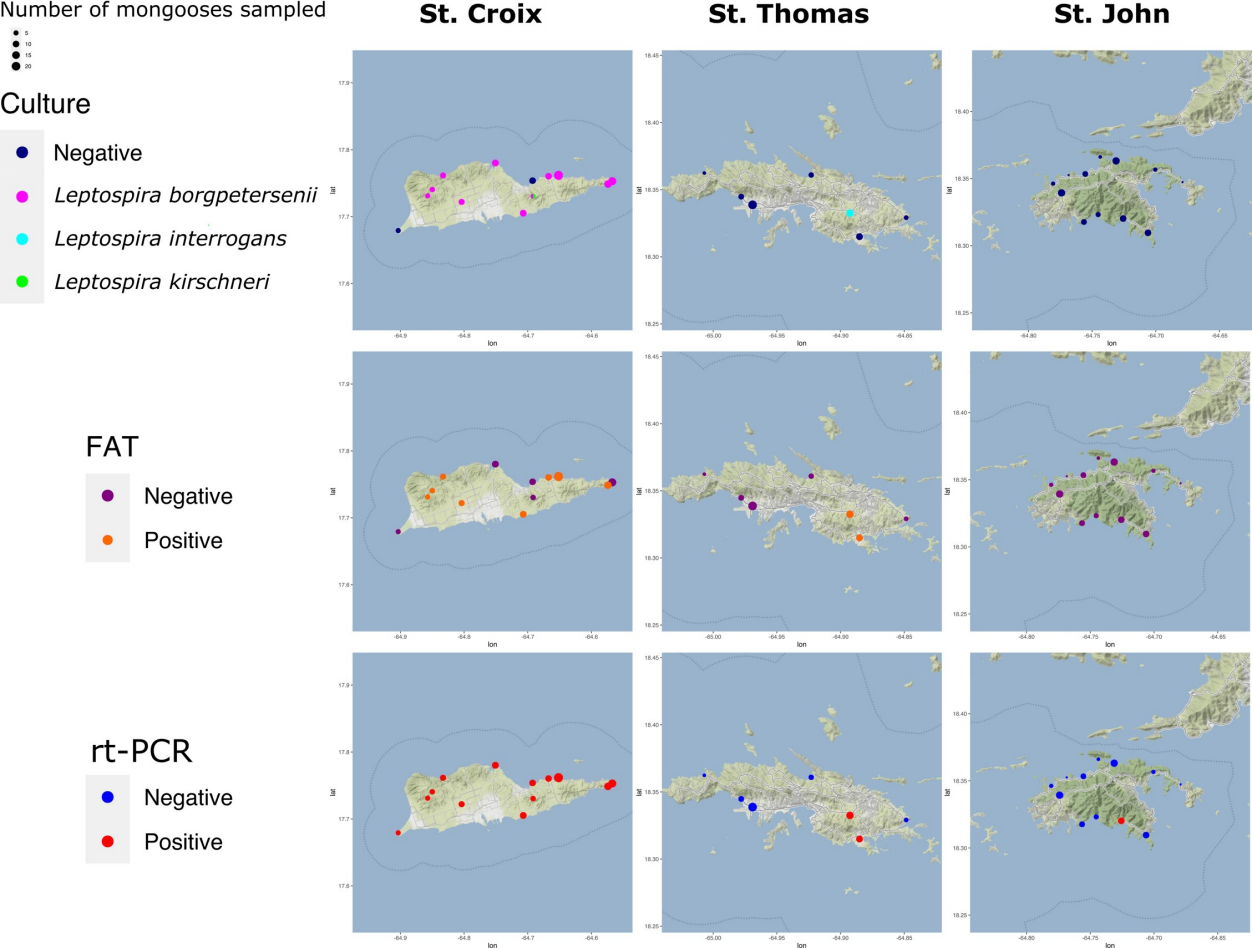
Sampling numbers and carriage (bacterial culture, FAT, rt-PCR) of *Leptospira* spp. in small Indian mongooses in the U.S. Virgin Islands^ab^. ^a^FAT: florescent antibody test; rt-PCR: Real time PCR. ^b^Map base layer available under CC BY 3.0 license: http://maps.stamen.com/terrain/#10/18.0114/-64.7823.

We sampled 274 mongooses across the three main USVI islands during August 2019–March 2020. Table 1 illustrates demographics and location of the mongooses sampled, whereas Fig 1 displays the location of trapping and laboratory results. We obtained mongooses from 31 sample sites (STX = 13; STT = 6; STJ = 12), and 8–54 mongooses were sampled from each region (n = 10) (S2 Table).

**Table 1:** Age, sex, and location of mongooses sampled (n = 274) for leptospirosis in the U.S. Virgin Islands.

Island		Female	Male	Total
**St. Croix** (n = 134)	**Adult**	54	66	120
	**Juvenile**	9	5	14
**St. Thomas** (n = 65)	**Adult**	26	27	53
	**Juvenile**	12	0	12
**St. John (**n = 75)	**Adult**	33	36	69
	**Juvenile**	5	1	6
	**Total**	139	135	274

Overall, leptospirosis seroprevalence among mongooses was 33.9% (87 out of 256 tested mongoose serum samples). Certain serum samples were not tested because of insufficient volume or poor quality (18/274). Of 202 reactions observed (S3 Table), serum was most reactive to the following serogroups: Sejroe (55.4%), Icterohaemorrhagiae (13.4%), Pyrogenes (7.4%), Mini (6.9%), Cynopteri (4.5%), Australis (4.0%), Hebdomadis (3.5%), Autumnalis (2.0%), Mankarso (1.5%), Pomona (1.0%), and Ballum (0.5%). In total, 27.6% of seropositive samples (24/87) had a high titer of ≥1:1600, including two samples collected from the same site (Prune Bay, St. Croix) that had exceptionally high titers reactive to Sejroe (1:6400 and 1:12800). Additionally, 36.8% (32/87) had a titer of 1:400–1:800, and 35.6% (31/87) had a titer of 1:100–1:200. Fifty-four of the 87 seropositive mongooses (62.1%) had antibodies to >1 serovar. An autochthonous strain recovered from bacterial culture of a single mongoose during this study was included in the MAT panel, species *Leptospira borgpetersenii*, serogroup Sejroe, serovar undetermined; this animal, identification number “LM31”, was sampled from Southgate on St. Croix. We detected serum reactivity to strain LM31 in 16/87 (18.4%) MAT positive samples. Comprehensive data for titers and associated serovar(s) of MAT-positive serum samples are in S3 Table.

**Fig 2:**
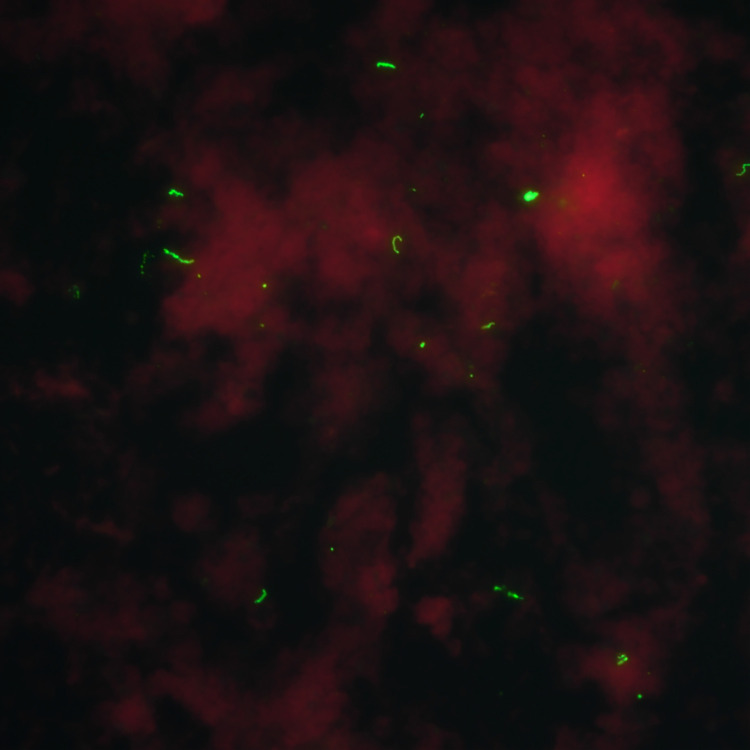
Positive florescent antibody test (FAT) image of a mongoose kidney from the U.S. Virgin Islands (final magnification x400)^a^. ^a^ Figure credit: Mr. Richard Hornsby, USDA-ARS.

**Fig 3:**
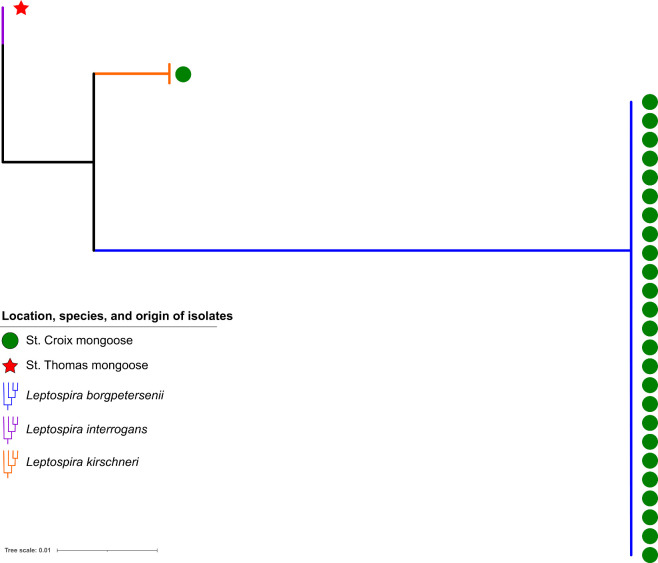
RAxML phylogenic tree of *Leptospira* isolates (n = 27) obtained from U.S.Virgin Islands mongooses using a *ppk* gene alignment. Most isolates were *L*. *borgpetersenii* retrieved from St. Croix mongooses (n = 25).

*Leptospira* isolates were obtained from 27 mongooses. Mongoose sampling sites indicating the highest isolate positivity rates were Estate Lower Love (4/8), Prune Bay (5/23), and Recovery Hill (4/6) on St. Croix; 3/27 culture-positive mongooses were juvenile, the remainder were adults. *Leptospira* spp. isolates were obtained from 26 mongooses on St. Croix, one mongoose isolate was obtained on St. Thomas. Of mongoose isolates, 25 were *L*. *borgpetersenii* by gene sequencing, one was *L*. *interrogans* (LM244), and one was *L*. *kirschneri* (LM157). All *L*. *borgpetersenii* were serotyped as belonging to serogroup Sejroe and the *L*. *interrogans* and *L*. *kirschneri* isolates were both serotyped as belonging to serogroup Icterohaemorrhagiae. Sera from the two mongooses infected with serogroup Icterohaemorrhagiae (LM157, LM244) was only reactive to serogroup Icterohaemorrhagiae (serovar Copenhageni) by MAT. Fig 2 and S1 Video capture imaging of FAT and culture positive specimens from mongooses. Fig 3 illustrates a RAxML phylogenetic tree of the 27 isolates with three distinct clades.

Of 274 mongoose kidney samples tested using rt-PCR, 34 (12.4%) were positive and indicated an active infection. Of 270 mongoose kidney samples tested for FAT, 16 (5.9%) were positive. The overall bacterial presence (positive on any of PCR, FAT, or culture) detected in the kidneys was 14.2% (39/274). Table 2 shows sample results for mongooses by island. Twelve (4.4%) of 274 cultures were contaminated and considered negative for calculations. We assessed how successful common diagnostic assays for *Leptospira* (e.g. rt-PCR, FAT, MAT) were in relation to the gold standard (bacterial culture). For isolation of *Leptospira* cultures, FAT sensitivity was low (52%), but positive predictive values (PPV) were high (88%); rt-PCR laboratory testing resulted in high sensitivity (81%), but lower PPV (65%); MAT had poor sensitivity (28%) and PPV (8%) (Table 3). S4 Table provides data for the calculations performed for Table 3 between mongoose kidney samples tested by culture, FAT, rt-PCR, and MAT stratified by test type. Calculation of intraclass correlation (*ρ*) estimates of each laboratory detection method compared to island and location, revealed weak correlation for bacterial culture (*ρ =* 0.25–0.27), while rt-PCR, MAT, and FAT had very weak correlation (Table 4).

**Table 2:** Mongoose serum and kidney sample test results using MAT, FAT, rt-PCR and culture by island (number positive/number tested)^a^.

Island	MAT	FAT	rt-PCR	Culture
**St. Croix**	74/130 (57%)	14/134 (10%)	28/134 (21%)	26/134 (19%)
**St. Thomas**	11/63 (18%)	2/64 (3%)	4/65 (6%)	1/65 (2%)
**St. John**	2/63 (3%)	0/72 (0%)	2/75 (3%)	0/75 (0%)
**Totals**	87/256 (34%)	16/270 (6%)	34/274 (12%)	27/274 (10%)

^a^MAT: microscopic agglutination test; FAT: florescent antibody test; rt-PCR: real time PCR

**Table 3:** Positive predictive values (PPV), negative predictive values (NPV), sensitivity, and specificity of MAT, FAT, and rt-PCR testing of mongoose serum and kidney samples for predicting succesful isolation of *Leptospira* spp. by culture in the U.S. Virgin Islands^a^^b^.

Test	PPV	NPV	Sensitivity	Specificity
**FAT**	88%	95%	52%	99%
**rt-PCR**	65%	98%	81%	95%
**MAT**	8%	66%	28%	89%

^a^MAT: microscopic agglutination test; FAT: florescent antibody test; rt-PCR: real time PCR

^b^Full data available in S4 Table

**Table 4:** Intraclass correlation coefficient (*ρ*) point estimates for *Leptospira* detections in mongooses, by island and location.

Laboratory method	Location (sampling site)	Island
MAT	0.19	0.04
FAT	0.02	0.004
rt-PCR	0.08	0.06
All Culture	0.27	0.25
*L*. *borgpetersenii* culture	0.25	0.72

## Discussion

Results of this investigation show evidence of *Leptospira* spp. exposure (MAT) and carriage (rt-PCR, FAT, or culture) in mongooses across the three islands of USVI. St. Croix mongooses had a much higher prevalence of leptospirosis, compared with those on St. Thomas and St. John (Table 2, Fig 1). The most likely explanation is the historical and current dominance of agriculture on St. Croix, compared to the other islands that could have led to the spillover introduction of *Leptospira* from livestock to mongooses. St. Croix has 135 farms, compared with just 40 on St. Thomas and 15 on St. John (B. Bradford, USVI Director of Veterinary Services, pers. comm).

The high number of isolates (n = 27) obtained in this investigation is significant because *Leptospira* spp. are fastidious aerobic bacteria, which can take months to observe. Although cultivation of leptospires is the reference standard for confirming leptospirosis diagnosis, sensitivity is low because of the difficulty of successfully growing the organism [27]. In our investigation, the positive predictive value of rt-PCR and FAT testing of mongoose samples for predicting *Leptospira* spp. isolation was 65% and 88%, respectively. Our successful recovery of leptospires reflects the use of multiple growth media, including the most recently developed HAN media which facilitates isolation and propagation of leptospires at 37°C compared to the conventional temperature of 28–30°C. Considering the poor sensitivity of MAT (28%), this type of diagnostic assay may have limited value in detecting *Leptospira* spp. in a reservoir host. Furthermore, these data suggest that multiple diagnostic assays should be employed to enhance chances of detection.

The observed seroprevalence level of 33.9% in small Indian mongooses in the USVI mirrors trends reported in previous studies of the same species in regions with similar topography and tropical conditions [12]. However, this seroprevalence might be an underestimation of the true exposure prevalence due to the lack of humoral response in mongooses despite carriage of *Leptospira* spp. in the kidneys. This biological equilibrium has been observed in other maintenance hosts such as rodents and could explain why the same mongooses that tested MAT seronegative could have positive resulting kidney samples (2 FAT-positive, 6 rt-PCR-positive, 1 bacterial culture-positive) [28–30]. Similarly, Rajeev et al. recently found that despite evidence of renal carriage, rodents in St. Kitts showed significantly less seroreactivity to *L*. *borgpetersenii* than *L*. *interrogans* [29]. Interestingly, the one bacterial culture-positive kidney with corresponding negative MAT serum from our data was *L*. *borgpetersenii*. These findings indicate that mongooses are reservoir hosts of this zoonotic disease as *Leptospira* sequester in the kidney following systemic infection via hematogenous spread after entering the host [28].

We compared our data with Shiokawa et al.’s 2018 mongoose survey of St. Kitts and found similar reactivity with the following *Leptospira* serovars: Autumnalis, Bratislava, Icterohaemorrhagiae, Mankarso, and Pomona [12]. Furthermore, when comparing these data with Ahl et al.’s 1992 study of antibody prevalence in sheep and goats on St. Croix, USVI, we found overlapping reaction with 11 serovars (Australis, Autumnalis, Ballum, Bratislava, Copenhageni, Hardjo, Hebdomadis, Pomona, Pyrogenes, Sejroe, and Wolffi) [7]. Moreover, 18.4% reactivity to the autochthonous strain, LM31, reinforces the need to explore for locally prevalent *Leptospira* isolates that should be included in the MAT panel used for investigative and diagnostic purposes in both animals and humans as described by Bourhy et al. [31].

Phylogenetic analysis of bacterial isolates revealed that the majority of St. Croix mongooses (96%) shared a similar clade within *L*. *borgpetersenii*, although two mongoose outliers were also detected (one on St. Croix and one on St. Thomas) (Fig 3). These findings suggest that although mongooses are likely a reservoir host for *L*. *borgpetersenii* on St. Croix, they might also be a spillover host for *L*. *interrogans* and *L*. *kirschneri*. Table 4 indicates that a strong correlation was exhibited for *L*. *borgpetersenii* by island (*ρ =* 0.72). While this finding is intuitive, since all *L*. *borgpetersenii* were retrieved on one island, the weak intraclass correlation analysis for location (*ρ =* 0.25) indicates that if one mongoose in a sampling site was a carrier for *Leptospira*, the other mongooses in the immediate vicinity were not guaranteed to also be carriers. The rare isolation of other *Leptospira* species (n = 2/27) suggests these are the result of spillover from other animal reservoirs; further evaluation of other animal reservoirs in the USVI, particularly rodents, will provide greater clarity to transmission dynamics among other species.

Limitations of our results include the cross-sectional study design sampling methodology, which could have led to potential sampling bias due to limited access to remote sampling sites and temporal variation over eight months. However, we sampled at least two study sites in each of ten geographic regions in the USVI to obtain a diverse sample set of mongooses throughout the territory.

## Conclusion

The USVI animal leptospirosis surveillance project identified mongooses as reservoirs and potential vectors of disease to the local population. Through this project, the invasive small Indian mongoose in USVI were found to be exposed to and harbor pathogenic *Leptospira* spp. adding to the limited data available concerning *Leptospira* spp. animal reservoirs of the Caribbean region. These findings highlight the potential role mongooses could play in the epidemiologic cycle of leptospirosis and the potential risk for exposure and infection to humans, domestic animals, and other species at risk. This project resulted in built capacity for the USVI to engage a wide range of collaborators for cohesive leptospirosis prevention and surveillance efforts. This partnership brought vital supplies, training, and equipment to continue monitoring for leptospirosis and building a laboratory network for transfer of samples for ongoing testing and surveillance.

Lastly, our findings demonstrate that FAT and rt-PCR testing have the most value for predicting bacterial carriage of *Leptospira* in kidneys, which could influence future diagnostics and research in this field. We were very encouraged at the ability to isolate *Leptospira*, a notoriously fastidious diderm bacteria, in Hornsby-Alt-Nally (HAN) medium from kidneys aseptically obtained in tropical field surgery conditions and shipped over 2000 miles from U.S. Virgin Islands to Ames, Iowa, USA. Bacterial isolation and whole genome sequencing of *Leptospira* provides the best method for source attribution of leptospirosis infections in humans and can help direct public health interventions towards potential animal reservoirs.

## Supporting information

S1 Table *Leptospira* serogroups and serovars in the microscopic agglutination testing (MAT) panel used by CDC-BSPB^a^ or USDA-NVSL^b^ in the testing of mongoose serum samples in the U.S. Virgin Islands. ^a^CDC-BSPD = Centers for Disease Control and Prevention Bacterial Special Pathogens Branch. ^b^USDA-NVSL = U.S. Department of Agriculture National Veterinary Service Laboratories.
(DOCX)

S2 Table Mongooses sampled (n = 274) for *Leptospira* spp. by region and sampling location—United States Virgin Islands, 2019–2020. (DOCX)

S3 Table Titer and associated serovar(s) of MAT (microscopic agglutination test) positive mongoose serum samples (n = 87/256) in the U.S. Virgin Islands; 202 reactions observed (St. Croix: STX; St. Thomas: STT, St. John: STJ). (DOCX)

S4 Table United States Virgin Islands mongoose bacterial kidney culture, FAT, rt-PCR, and MAT test results stratified by test method^a^. ^a^FAT: florescent antibody test; rt-PCR: Real time PCR; MAT: microscopic agglutination test.
(DOCX)

S1 Video Culture positive video (40X) of a mongoose kidney from the U.S. Virgin Islands in Hornsby-Alt-Nally (HAN) media postinoculation^a^.^a^ Video credit: Mr. Richard Hornsby, USDA-ARS. (MP4)
