# Supplementary material for: Mongooses (Urva auropunctata) as reservoir hosts of Leptospira species in the United States Virgin Islands, 2019–2020
Source: PLoS Negl Trop Dis. 2021 Nov 15;15(11):e0009859. doi: 10.1371/journal.pntd.0009859 (PMC8592401; doi:10.1371/journal.pntd.0009859)
Supplement: S1 Table — aCDC-BSPD = Centers for Disease Control and Prevention Bacterial Special Pathogens Branch. bUSDA-NVSL = U.S. Department of Agriculture National Veterinary Service Laboratories. (DOCX) [file pntd.0009859.s001.docx]

**S1 Table. *Leptospira* serogroups and serovars in the microscopic agglutination testing (MAT) panel used by CDC-BSPB^a^ or USDA-NVSL^b^ in the testing of mongoose serum samples in the U.S. Virgin Islands**

|  | **Serogroup** | **Serovar** |
| --- | --- | --- |
| **CDC-BSPB** |  |  |
| 1. | Celledoni | Celledoni |
| 2. | Hebdomadis | Borincana |
| 3. | Mankarso | Mankarso |
| 4. | Javanica | Javanica |
| 5. | Mini | Georgia |
| 6. | Pyrogenes | Alexi |
| 7. | Sejroe | Wolffi |
| 8. | Icterohaemorrhagiae | Icterohaemorrhagiae |
| **USDA-NVSL** |  |  |
| 1. | Sejroe | Hardjo |
| 2. | Sejroe | Sejroe |
| 3. | Hebdomadis | Hebdomadis |
| 4. | Mini | Szwajizak |
| 5. | Icterohaemorrhagiae | Copenhageni |
| 6. | Sejroe | Undetermined (LM31)^c^ |
| **Both CDC-BSPB and USDA-NVSL** |  |  |
| 1. | Australis | Australis |
| 2. | Autumnalis | Autumnalis |
| 3. | Ballum | Ballum |
| 4. | Bataviae | Bataviae |
| 5. | Canicola | Canicola |
| 6. | Grippotyphosa | Grippotyphosa |
| 7. | Pomona | Pomona |
| 8. | Pyrogenes | Pyrogenes |
| 9. | Australis | Bratislava |
| 10. | Cynopteri | Cynopteri |
| 11. | Djasiman | Djasiman |
| 12. | Tarassovi | Tarassovi |

^a^CDC-BSPD = Centers for Disease Control and Prevention Bacterial Special Pathogens Branch.

^b^USDA-NVSL = U.S. Department of Agriculture National Veterinary Research Laboratories.

^c^Autochthonous strain recovered from mongoose “LM31” on St. Croix, U.S. Virgin Islands, species *Leptospira borgpetersenii*, serogroup Sejroe, serovar undetermined.
