# Supplementary material for: Mongooses (Urva auropunctata) as reservoir hosts of Leptospira species in the United States Virgin Islands, 2019–2020
Source: PLoS Negl Trop Dis. 2021 Nov 15;15(11):e0009859. doi: 10.1371/journal.pntd.0009859 (PMC8592401; doi:10.1371/journal.pntd.0009859)
Supplement: S2 Table — (DOCX) [file pntd.0009859.s002.docx]

**S2 Table. Mongooses sampled (n=274) for *Leptospira* spp. by region and sampling location — United States Virgin Islands, 2019–2020**

| **Island** | **Region** | **Sampling Location** | **Number of mongooses sampled** |
| --- | --- | --- | --- |
| St. Croix (STX)  (n = 134) | West  (Zone 1)  (n = 24) | Carambola | 8 |
|  |  | Jolly_Hill | 5 |
|  |  | Montpellier | 6 |
|  |  | Sandy_Point | 5 |
|  | Central  (Zone 2)  (n = 30) | Lower_Love | 8 |
|  |  | Haypenny_Beach | 11 |
|  |  | Salt_River | 11 |
|  | East  (Zone 3)  (n = 26) | Altona_Lagoon | 10 |
|  |  | Recovery_Hill | 6 |
|  |  | Southgate | 10 |
|  | Far East  (Zone 4)  (n = 54) | East_End_Bay | 17 |
|  |  | Issacs_Bay | 14 |
|  |  | Prune_Bay | 23 |
| St. Thomas (STT)  (n = 65) | West  (Zone 5)  (n = 8) | Magens_Bay | 6 |
|  |  | Stumpy_Bay | 2 |
|  | Central  (Zone 6)  (n = 28) | Airport | 21 |
|  |  | Brewers Bay | 7 |
|  | East  (Zone 7)  (n = 29) | Bovoni_Landfill | 11 |
|  |  | Tutu | 14 |
|  |  | Red_Hook_Point | 4 |
| St. John (STJ)  (n = 75) | West  (Zone 8)  (n = 31) | Gifft_Hill_Landfill | 13 |
|  |  | Hawksnest_Beach | 3 |
|  |  | Trunk_Bay | 1 |
|  |  | Western_Reef | 7 |
|  | Central  (Zone 9)  (n = 17) | Cinnamon_Bay | 7 |
|  |  | Francis_Bay | 2 |
|  |  | Lameshur_Bay | 10 |
|  |  | Reef_Bay | 5 |
|  | East  (Zone 10)  (n = 27) | Annaberg_Plantation | 13 |
|  |  | Brown_Bay | 3 |
|  |  | Haulover_Bay | 1 |
|  |  | Salt_Pond | 10 |
