# Supplementary material for: Mongooses (Urva auropunctata) as reservoir hosts of Leptospira species in the United States Virgin Islands, 2019–2020
Source: PLoS Negl Trop Dis. 2021 Nov 15;15(11):e0009859. doi: 10.1371/journal.pntd.0009859 (PMC8592401; doi:10.1371/journal.pntd.0009859)
Supplement: S3 Table — (DOCX) [file pntd.0009859.s003.docx]

**S3 Table.** **Titer and associated serovar(s) of MAT (microscopic agglutination test) positive mongoose serum samples (n = 87/256) in the U.S. Virgin Islands; 202 reactions observed (St. Croix: STX; St. Thomas: STT, St. John: STJ)**

| **Island** | **LM#** | **ALX^a^** | **AUS** | **AUT** | **BAL** | **BAT** | **BOR** | **BRA** | **CAN** | **CEL** | **COP** | **CYN** | **DJA** | **GEO** | **GRI** | **HAR** | **HEB** | **ICT** | **JAV** | **MAN** | **MIN** | **POM** | **PYR** | **SEJ** | **TAR** | **WOL** | **LM31** |
| --- | --- | --- | --- | --- | --- | --- | --- | --- | --- | --- | --- | --- | --- | --- | --- | --- | --- | --- | --- | --- | --- | --- | --- | --- | --- | --- | --- |
| **STX** | 8 | X^b^ | X | X | X | X | X | X | X | X | X | X | X | X | X | - | - | X | X | X | - | X | X | - | X | 200 | - |
|  | 11 | X | X | X | X | X | X | X | X | X | X | X | X | X | X | - | - | 100 | X | X | - | X | X | - | X | 200 | - |
|  | 23 | X | X | X | X | X | X | X | X | X | X | X | X | X | X | - | - | X | X | X | - | X | X | - | X | 400 | - |
|  | 26 | X | X | X | X | X | X | X | X | X | X | X | X | X | X | - | - | X | X | X | - | X | X | - | X | 200 | - |
|  | 31 | X | X | X | X | X | X | X | X | X | X | X | X | X | X | - | - | 200 | X | 100 | - | X | X | - | X | 100 | - |
|  | 63 | X | X | X | X | X | X | X | X | X | X | X | X | X | X | - | - | X | X | X | - | X | X | - | X | 800 | - |
|  | 64 | X | X | X | X | X | X | X | X | X | X | X | X | X | X | - | - | X | X | X | - | X | X | - | X | 100 | X |
|  | 66 | X | X | X | X | X | X | X | X | X | X | X | X | X | X | - | - | X | X | X | - | X | X | - | X | 400 | X |
|  | 68 | X | 200 | 800 | X | X | X | 800 | X | X | X | 400 | X | 400 | X | - | - | 200 | X | 800 | - | X | X | - | X | 1600 | 200 |
|  | 72 | X | X | X | X | X | X | X | X | X | X | X | X | X | X | - | - | X | X | X | - | X | X | - | X | 100 | X |
|  | 73 | X | X | X | X | X | X | X | X | X | X | X | X | X | X | - | - | X | X | X | - | X | X | - | X | 200 | 100 |
|  | 75 | X | X | X | X | X | X | X | X | X | X | X | X | X | X | - | - | X | X | X | - | X | X | - | X | 400 | X |
|  | 77 | X | X | X | X | X | X | X | X | X | X | X | X | X | X | - | - | 200 | X | 200 | - | X | X | - | X | X | X |
|  | 82 | X | X | X | X | X | X | X | X | X | X | X | X | X | X | - | - | X | X | X | - | X | 100 | - | X | 100 | X |
|  | 84 | X | X | X | X | X | X | X | X | X | X | X | X | X | X | - | - | X | X | X | - | X | X | - | X | 100 | 100 |
|  | 86 | X | X | X | X | X | X | X | X | X | X | X | X | X | X | - | - | X | X | X | - | X | X | - | X | 200 | X |
|  | 91 | X | X | X | X | X | X | X | X | X | X | X | X | 100 | X | - | - | X | X | X | - | X | X | - | X | 3200 | X |
|  | 92 | X | X | X | X | X | X | X | X | X | X | X | X | X | X | - | - | X | X | X | - | X | X | - | X | 100 | 100 |
|  | 93 | X | X | X | X | X | X | X | X | X | X | X | X | X | X | - | - | X | X | X | - | X | X | - | X | 800 | X |
|  | 94 | - | X | X | X | X | - | X | X | - | X | X | X | - | X | X | X | - | - | - | X | X | X | 100 | X | - | X |
|  | 95 | - | X | X | X | X | - | X | X | - | X | X | X | - | X | 100 | X | - | - | - | X | X | X | 800 | X | - | X |
|  | 96 | - | X | X | X | X | - | X | X | - | X | X | X | - | X | X | X | - | - | - | X | X | X | 400 | X | - | X |
|  | 99 | - | X | X | X | X | - | X | X | - | X | X | X | - | X | 400 | X | - | - | - | X | X | X | 3200 | X | - | X |
|  | 102 | - | X | X | X | X | - | X | X | - | X | X | X | - | X | 200 | X | - | - | - | X | X | X | 800 | X | - | X |
|  | 103 | - | X | X | X | X | - | X | X | - | X | X | X | - | X | 1600 | 200 | - | - | - | 800 | X | X | 12800 | X | - | 100 |
|  | 104 | - | X | X | X | X | - | X | X | - | X | X | X | - | X | 800 | 100 | - | - | - | 400 | X | X | 6400 | X | - | 100 |
|  | 105 | - | X | X | X | X | - | X | X | - | X | X | X | - | X | 100 | X | - | - | - | X | X | X | 400 | X | - | X |
|  | 107 | - | X | X | X | X | - | X | X | - | X | X | X | - | X | X | X | - | - | - | X | X | X | 800 | X | - | X |
|  | 108 | - | X | X | X | X | - | X | X | - | X | X | X | - | X | 200 | X | - | - | - | X | X | X | 3200 | X | - | X |
|  | 111 | - | X | X | X | X | - | X | X | - | 800 | X | X | - | X | X | X | - | - | - | X | X | 400 | X | X | - | X |
|  | 112 | - | X | X | X | X | - | X | X | - | X | X | X | - | X | 200 | X | - | - | - | X | X | X | 800 | X | - | 100 |
|  | 114 | - | X | X | X | X | - | X | X | - | X | X | X | - | X | 1600 | X | - | - | - | X | X | X | 1600 | X | - | X |
|  | 116 | - | X | X | X | X | - | X | X | - | 3200 | X | X | - | X | X | X | - | - | - | X | X | 800 | X | X | - | X |
|  | 118 | - | X | X | X | X | - | X | X | - | X | 100 | X | - | X | X | X | - | - | - | X | X | X | 200 | X | - | X |
|  | 119 | - | X | X | X | X | - | X | X | - | 800 | 100 | X | - | X | 400 | X | - | - | - | X | X | 200 | 1600 | X | - | 100 |
|  | 120 | - | X | X | X | X | - | X | X | - | X | 100 | X | - | X | 800 | X | - | - | - | X | X | X | 3200 | X | - | 100 |
|  | 122 | - | X | X | X | X | - | X | X | - | X | 100 | X | - | X | X | X | - | - | - | X | X | X | X | X | - | X |
|  | 123 | - | X | X | X | X | - | X | X | - | X | 200 | X | - | X | X | X | - | - | - | X | X | X | X | X | - | X |
|  | 124 | - | X | X | X | X | - | X | X | - | 1600 | X | X | - | X | 800 | 100 | - | - | - | 100 | X | 400 | 1600 | X | - | X |
|  | 126 | - | X | X | X | X | - | 100 | X | - | X | X | X | - | X | X | X | - | - | - | X | X | X | X | X | - | X |
|  | 127 | - | X | X | X | X | - | X | X | - | X | 100 | X | - | X | 100 | X | - | - | - | X | X | X | 400 | X | - | X |
|  | 129 | - | X | X | X | X | - | X | X | - | X | X | X | - | X | 400 | X | - | - | - | 200 | X | X | 1600 | X | - | 100 |
|  | 130 | - | X | X | X | X | - | X | X | - | X | X | X | - | X | X | X | - | - | - | X | X | X | 400 | X | - | 100 |
|  | 131 | - | X | X | X | X | - | 200 | X | - | 100 | X | X | - | X | X | X | - | - | - | X | X | X | 400 | X | - | 100 |
|  | 132 | - | X | X | X | X | - | X | X | - | X | X | X | - | X | 800 | X | - | - | - | X | X | X | 800 | X | - | X |
|  | 133 | - | X | X | X | X | - | X | X | - | X | X | X | - | X | 800 | X | - | - | - | 100 | X | X | 1600 | X | - | X |
|  | 134 | - | X | X | X | X | - | X | X | - | 200 | X | X | - | X | X | X | - | - | - | X | X | X | X | X | - | X |
|  | 135 | - | X | X | X | X | - | X | X | - | X | X | X | - | X | 200 | X | - | - | - | X | X | X | 400 | X | - | X |
|  | 140 | - | X | X | X | X | - | X | X | - | 400 | X | X | - | X | X | X | - | - | - | X | X | 200 | X | X | - | X |
|  | 141 | - | X | X | X | X | - | X | X | - | X | X | X | - | X | 800 | X | - | - | - | 200 | X | X | 800 | X | - | X |
|  | 147 | - | X | X | X | X | - | X | X | - | X | X | X | - | X | 100 | X | - | - | - | X | X | X | 400 | X | - | X |
|  | 150 | - | X | X | X | X | - | X | X | - | X | X | X | - | X | 800 | X | - | - | - | X | X | X | 3200 | X | - | 100 |
|  | 152 | - | X | X | X | X | - | X | X | - | 800 | X | X | - | X | 800 | X | - | - | - | X | X | 400 | 1600 | X | - | 100 |
|  | 153 | - | X | X | X | X | - | X | X | - | X | X | X | - | X | 400 | X | - | - | - | X | X | X | 1600 | X | - | X |
|  | 154 | - | X | X | X | X | - | X | X | - | X | X | X | - | X | 100 | 200 | - | - | - | 100 | X | X | 800 | X | - | X |
|  | 155 | - | X | X | X | X | - | X | X | - | X | X | X | - | X | X | X | - | - | - | X | X | X | 400 | X | - | 100 |
|  | 156 | - | X | X | X | X | - | X | X | - | 3200 | X | X | - | X | 400 | X | - | - | - | X | X | 400 | 3200 | X | - | 100 |
|  | 157 | - | X | X | X | X | - | X | X | - | 800 | X | X | - | X | X | X | - | - | - | X | X | X | X | X | - | X |
|  | 158 | - | X | X | X | X | - | X | X | - | X | X | X | - | X | X | X | - | - | - | X | X | X | 800 | X | - | X |
|  | 160 | - | X | X | X | X | - | X | X | - | 800 | X | X | - | X | 200 | X | - | - | - | X | X | 800 | 800 | X | - | X |
|  | 161 | - | X | X | X | X | - | X | X | - | 200 | X | X | - | X | 400 | X | - | - | - | 200 | X | 100 | 1600 | X | - | X |
|  | 162 | - | X | X | X | X | - | X | X | - | X | X | X | - | X | 1600 | 400 | - | - | - | 800 | X | 100 | 1600 | X | - | X |
|  | 164 | - | X | X | X | X | - | X | X | - | X | X | X | - | X | X | X | - | - | - | X | X | X | 200 | X | - | X |
|  | 165 | - | X | X | X | X | - | X | X | - | X | X | X | - | X | 400 | 100 | - | - | - | 200 | X | X | 3200 | X | - | X |
|  | 166 | - | X | X | X | X | - | X | X | - | X | X | X | - | X | X | X | - | - | - | X | X | X | 800 | X | - | X |
|  | 167 | - | X | X | X | X | - | X | X | - | X | X | X | - | X | X | X | - | - | - | X | X | X | 100 | X | - | X |
|  | 297 | - | X | X | X | X | - | X | X | - | 400 | X | X | - | X | X | X | - | - | - | X | X | 100 | X | X | - | X |
|  | 298 | - | X | X | X | X | - | X | X | - | X | X | X | - | X | X | X | - | - | - | X | X | X | 800 | X | - | X |
|  | 300 | - | X | X | X | X | - | X | X | - | 800 | X | X | - | X | X | X | - | - | - | X | X | 400 | X | X | - | X |
|  | 302 | - | X | X | X | X | - | X | X | - | X | X | X | - | X | 100 | X | - | - | - | X | X | X | 800 | X | - | X |
|  | 307 | - | X | X | X | X | - | X | X | - | X | X | X | - | X | 400 | X | - | - | - | X | X | X | 1600 | X | - | X |
|  | 308 | - | X | X | X | X | - | X | X | - | X | X | X | - | X | X | X | - | - | - | X | X | X | 200 | X | - | X |
|  | 310 | - | X | X | X | X | - | X | X | - | 200 | X | X | - | X | 400 | X | - | - | - | 100 | X | 100 | 800 | X | - | X |
|  | 311 | - | X | X | X | X | - | X | X | - | X | X | X | - | X | 3200 | 100 | - | - | - | 400 | X | X | 3200 | X | - | X |
|  |  |  |  |  |  |  |  |  |  |  |  |  |  |  |  |  |  |  |  |  |  |  |  |  |  |  |  |
| **STT** | 171 | - | X | X | X | X | - | X | X | - | X | X | X | - | X | X | X | - | - | - | X | X | X | 100 | X | - | X |
|  | 181 | - | X | X | 100 | X | - | X | X | - | X | X | X | - | X | X | X | - | - | - | X | X | X | X | X | - | X |
|  | 195 | - | X | X | X | X | - | X | X | - | 100 | X | X | - | X | X | X | - | - | - | X | X | X | X | X | - | X |
|  | 225 | - | X | 200 | X | X | - | 3200 | X | - | 400 | X | X | - | X | X | X | - | - | - | X | 100 | X | X | X | - | X |
|  | 226 | - | X | X | X | X | - | X | X | - | 100 | X | X | - | X | X | X | - | - | - | X | X | X | X | X | - | X |
|  | 228 | - | X | X | X | X | - | X | X | - | 200 | X | X | - | X | X | X | - | - | - | X | X | X | X | X | - | X |
|  | 230 | - | X | X | X | X | - | X | X | - | X | 100 | X | - | X | X | X | - | - | - | X | X | X | X | X | - | X |
|  | 243 | - | X | X | X | X | - | X | X | - | 200 | X | X | - | X | X | X | - | - | - | X | X | 200 | X | X | - | X |
|  | 244 | - | X | X | X | X | - | X | X | - | 200 | X | X | - | X | X | X | - | - | - | X | X | X | X | X | - | X |
|  | 245 | - | X | 200 | X | X | - | 800 | X | - | 200 | X | X | - | X | X | X | - | - | - | X | 400 | X | X | X | - | X |
|  | 248 | - | X | 200 | X | X | - | 3200 | X | - | 100 | X | X | - | X | X | X | - | - | - | X | X | X | X | X | - | X |
|  |  |  |  |  |  |  |  |  |  |  |  |  |  |  |  |  |  |  |  |  |  |  |  |  |  |  |  |
| **STJ** | 182 | - | X | X | X | X | - | 200 | X | - | X | X | X | - | X | X | X | - | - | - | X | X | X | X | X | - | X |
|  | 215 | - | X | X | X | X | - | X | X | - | X | 100 | X | - | X | X | X | - | - | - | X | X | X | X | X | - | X |

^a^ALX = Pyrogenes, sv Alexi; AUS = Australis, serovar (sv) Australis; AUT = Autumnalis, sv Autumnalis; BAL = Ballum, sv Ballum; BAT = Bataviae, sv Bataviae; BOR = Hebdomadis, sv Borincana; BRA = Australis, sv Bratislava; CAN = Canicola, sv Canicola; CEL = Celledoni, sv Celledoni; COP = Icterohaemorrhagiae, sv Copenhageni; CYN = Cynopteri, sv Cynopteri; DJA = Djasiman, sv Djasiman; GEO = Mini, sv Georgia; GRI = Grippotyphosa, sv Grippotyphosa; HAR = Sejroe, sv Hardjo; HEB = Hebdomadis, sv Hebdomadis; ICT = Icterohaemorrhagiae, sv Icterohaemorrhagiae; JAV = Javanica, sv Javanica; MAN = Mankarso, sv Mankarso; MIN = Mini, sv Szwajizak; POM = Pomona, sv Pomana; PYR = Pyrogenes, sv Pyrogenes; SEJ = Sejroe, sv Sejroe; TAR = Tarassovi, sv Tarassovi; WOL = Sejroe, sv Wolffi; LM31 = Autochthonous strain recovered from a mongoose on St. Croix, U.S. Virgin Islands, species *Leptospira borgpetersenii*, serogroup Sejroe, serovar undetermined.

^b^”X” = <1:100; “-“ = not tested
