# Supplementary material for: Mongooses (Urva auropunctata) as reservoir hosts of Leptospira species in the United States Virgin Islands, 2019–2020
Source: PLoS Negl Trop Dis. 2021 Nov 15;15(11):e0009859. doi: 10.1371/journal.pntd.0009859 (PMC8592401; doi:10.1371/journal.pntd.0009859)
Supplement: S4 Table — aFAT: florescent antibody test; rt-PCR: Real time PCR; MAT: microscopic agglutination test. (DOCX) [file pntd.0009859.s004.docx]

**S4 Table**. **United States Virgin Islands mongoose bacterial kidney culture, FAT, rt-PCR, and MAT test results stratified by test method^a^**

| **Test method** | | **Culture** | | | **FAT** | | | **rt-PCR** | | |
| --- | --- | --- | --- | --- | --- | --- | --- | --- | --- | --- |
|  |  | Positive | Negative | Total | Positive | Negative | Total | Positive | Negative | Total |
| FAT | Positive | 14 | 2 | 16 | - | - | - | - | - | - |
|  | Negative | 13 | 241 | 254 | - | - | - | - | - | - |
|  | Total | 27 | 243 | 270 | - | - | - | - | - | - |
| rt-PCR | Positive | 22 | 12 | 34 | 16 | 18 | 34 | - | - | - |
|  | Negative | 5 | 235 | 240 | 0 | 236 | 236 | - | - | - |
|  | Total | 27 | 247 | 274 | 16 | 254 | 270 | - | - | - |
| MAT | Positive | 7 | 79 | 86 | 14 | 73 | 87 | 27 | 60 | 87 |
|  | Negative | 18 | 152 | 170 | 2 | 163 | 165 | 6 | 163 | 169 |
|  | Total | 25 | 231 | 256 | 16 | 254 | 252 | 33 | 223 | 256 |

^a^FAT: florescent antibody test; rt-PCR: Real time PCR; MAT: microscopic agglutination test
